# Development and validation of the participatory approach questionnaire (*PARTAQUE*) in cancer teams: a ready-to-use tool for enhancing the quality of work life for caregivers

**DOI:** 10.3389/fpsyg.2025.1575442

**Published:** 2025-06-23

**Authors:** Julien Lejeune, Anaïs Galy, Evelyne Fouquereau, Christine Jeoffrion, Julia Aubouin-Bonnaventure, Hélène Coillot, Jimmy Bordarie, Romuald Seizeur, Philippe Colombat

**Affiliations:** ^1^QualiPsy UR 1901, Psychology Department, University of Tours, Tours, France; ^2^Service d'onco-hématologie pédiatrique, CHRU of Tours, Tours, France; ^3^Pôle Santé HEC Montréal, Department of Human Resources, HEC Montréal, Montréal, QC, Canada; ^4^LIP/PC2S, Université de Grenoble Alpes, Université Savoie Mont Blanc, Grenoble, France; ^5^EE 1901 Quality of Life and Psychological Health, Université de Tours, Tours, France; ^6^Service de Neurochirurgie, CHU Brest, Brest, France

**Keywords:** Participative Approach (PA), cancer teams, scale validation, quality of work life, quality of care

## Abstract

The Participative Approach (PA) has demonstrated the potential to enhance both the quality of work life and the quality of care within French cancer teams. Whilst a number of studies have demonstrated that the participatory approach exerts a positive impact on all stakeholders within the care context, none of them have thus far used a measurement of the PA that has been scientifically validated. Therefore, the aim of this research was to develop and validate the PARTAQUE (Participatory Approach Questionnaire) and to assess its psychometric properties. This objective was pursued by conducting three independent studies in French healthcare organizations. The assessment of the factorial structure of the scale confirmed a good fit with a second-order factor model, and the test of convergent and predictive validity showed consistent relationships with theorized outcomes (e.g., attitudinal and behavioral work outcomes, occupational health indicators). The implications for research and practice are then discussed.

## 1 Introduction

Working in oncology department is a daily challenge for all caregivers. Indeed, both the intensification (e.g., increased patient load) and the complexification of oncological care demands (e.g., wider variety of care protocols; increased use of sophisticated care technologies) exert chronic pressures on cancer team's members (Takvorian et al., 2020; Attieh et al., 2024). Accordingly, caregivers evolving in this type of occupational setting face acute risk of health-related problems in the workplace (e.g., burnout) (Smith et al., 2018; Colombat, 2020).

However, as highlighted by numerous studies, these observations are not solution-less (Attieh et al., 2024; Colombat et al., 2019; Chevalier et al., 2017; Lejeune et al., 2020). For example, relying on a team composed of multidisciplinary practitioners appears to be an interesting avenue to address these challenges, as a qualitative collaborative work environment is now recognized as a key antecedent for both the quality of work life of the caregivers and the quality of care (Lejeune et al., 2020, 2021).

In the USA, magnet hospitals have been developed to improve the working conditions and enhance the psychological wellbeing of caregivers. These establishments implement organizational and managerial measures based on eight essential dimensions: a patient-centered culture, caregiver expertise, support for training, transformational leadership, participative management, a collegial doctor/caregiver climate, clinical autonomy, and adequate workforce management. The implementation of the Magnet hospital model has been demonstrated to be associated with improved caregiver psychological health and reduced mortality (McHugh et al., 2013), along with enhanced patient-perceived quality of care (Stimpfel et al., 2016).

In France, the Participatory Approach (PA) is grounded in a different perspective. Indeed, it was initially developed by caregivers for caregivers—working in palliative departments (Bauchetet et al., 2011; Colombat et al., 2018; Colombat, 2020) and thus, do not rely on an hospital-driven initiative. Since its initial implementations in the late 1990s it has evolved into a distinctive work-organization model within the field of healthcare (Colombat, 2020; Lejeune et al., 2020, 2021). In this context, the PA emerges as a noteworthy collaborative opportunity which is now recognized on a national scale, as a tried-and-tested French model of department organization supported by the Ministry of Health and legally mandatory in all palliative care services since 2004.^1^

While magnet hospitals and the PA share numerous common features, including a patient-centered approach to decision-making, collaborative teamwork, and comprehensive caregiver training, aimed at enhancing patient care, a fundamental distinction emerges. Magnet hospitals are embedded within the organizational/managerial framework of the institution, whereas the PA emerges from the initiative of the head of department. Accordingly, the PA is not a part of an overarching institutional policy of the healthcare organization.

The PA is predicated on two fundamental lines of action, that is: enhancing team communication and implementing a project approach. To accomplish the aforementioned objectives, the PA is based on 4 key components, (1) multi-professional team meetings, (2) in-service training, (3) team support meetings, and (4) project approach. The following lines provide a detailed explanation of the different PA components.

First, team communication is developed through various tools, such as multi-professional team meetings, in-service training and team support meetings. Multidisciplinary team meetings are held to facilitate interdisciplinary discussion among health care providers working within the department. The purpose of these meetings is to ensure the comprehensive management of patients. In addition, in-service training ensures that all members of the care team (e.g., physicians, nurses, and nursing assistants) receive comparable and consistent occupational training. It provides opportunities for the team members to share their experiences, specifically for those who do not often express themselves. Furthermore, it is conducive to the establishment of qualitative interprofessional relationships, which are a critical component of team-building strategies (Bauchetet et al., 2020). Team support meetings, overseen by a psychotherapist, are designed to provide debriefing for care staff confronted with crisis situations.

Second, the project-based approach entails the identification of a problem or need and the establishment of interdisciplinary working groups to propose and discuss methodologies for addressing it. This approach can be used not only to manage a specific problem, but also as a global service team project. It enables caregivers to voice their concerns and participate autonomously in identifying and implementing solutions in their department.

Although several studies have examined the positive effects of the PA on the health at work of caregivers and quality of care (Chevalier et al., 2017; Colombat et al., 2018; Lejeune et al., 2020, 2021), none of these studies had at that time a validated scale of the PA for their demonstration. In fact, these studies mainly focused on examining various positive consequences of the PA (e.g., recognition at work, work support, job satisfaction) and their effects on different occupational outcomes, such as quality of work life and work performance indicators (e.g., quality of care). Notwithstanding the valuable contributions made to the extant knowledge base concerning the management of oncology teams, these studies did not rely on a scientifically validated measurement of the PA and its components. This could be problematic as it raises concerns about the psychometric qualities (e.g., convergent validity) of the measures used in these studies and hinders the ability to implement scientifically sound interventions for the teams involved in this research. Indeed, relying on a scientifically validated measure of the PA could be the occasion to highlight its benefits following its implementation in a team. Also, managers of the oncology team could rely on this valid measure to make informed decision and allocate resources where they are the most needed.

Accordingly, the present research focuses on the development and validation of the Participatory Approach Questionnaire (PARTAQUE), a scale designed to elicit the perceptions of care team members with regard to the degree of implementation of both the PA and its respective components. To this end, we followed a rigorous scale development and validation protocol, which involved an initial qualitative research (e.g., literature review, semi-structured interviews with healthcare professionals) to generate the item pool, followed by in-depth statistical analyses to test the factorial structure and validity of the scale (e.g., exploratory factor analysis—EFA; confirmatory factor analysis—CFA; convergent and predictive validity). All the steps of the development and validation protocol are described in detail in the different studies presented in the following sections.

## 2 Method

### 2.1 Item screening and questionnaire development

To develop a preliminary pool of items (Hinkin, 1998; Clark and Watson, 2019), we started by carrying out a broad review of the relevant literature to cover the field of the specified domain (i.e., participative approach).

Moreover, to enrich our understanding of the participatory approach, we also conducted face-to-face semi-structured interviews, which lasted between 30 min and an hour, with 21 healthcare professionals working in four hospitals and seven departments (i.e., medical oncology, hematology, pediatric onco-hematology, post-emergency internal medicine, pediatric onco-hemato-immunology, pediatric oncology, acute hospitalization) who had implemented a participative approach (Arnaudeau et al., 2024). Interviews were conducted by one of the researchers on the research team, and the interview guide contained questions about the four dimensions of participative approach (i.e., in-house training, team support, multi-professional meetings, project approach). The sample was composed of 7 nurses, 7 health managers, 5 care assistants and 2 nursery assistants. Among participants, 20 were women, the average age was 38 (SD = 9.6), and the average number of years of professional experience was 10.5 (SD = 6.6).

Therefore, using items found in scientific literature and verbatims from interviews, we developed six items for the in-house training dimension (sample items: “*You regularly benefit from in-house training*,” “*All caregivers have access to in-house training courses*”); six items for the team support dimension (sample items: “*All healthcare staff can take part in team support meetings*,” “*Team support meetings are held on a regular basis”*); seven items for the multi-professional meetings dimension (sample items: “*During multi-professional staff meetings, each participant has the opportunity to express his or her ideas*,” “*Decisions taken during multi-professional staff meetings are regularly implemented*”); and seven items for the project approach dimension (sample items: “*In these working groups, the subjects dealt with correspond to concrete professional concerns*,” “*The recommendations of these working groups are validated by the team”*). Thus, the initial version of the PARTAQUE contained 26 items.

### 2.2 Study 1: exploratory factor analysis of the PARTAQUE

The aim of Study 1 was to test the factorial structure and psychometric qualities of the PARTAQUE. More precisely, we carried out an Exploratory Factor Analysis (EFA) and checked its internal consistency.

#### 2.2.1 Participants

The sample consisted of 335 French healthcare professionals (59 men and 276 women), including 114 nurses (34.03%), 74 physicians (22.09%), 66 care assistants (19.70%), 43 health managers (12.84%), 5 hospital domestic services aide (1.49%), and 33 other healthcare professionals (9.85%). Their average age was 41.56 years (*SD* = 10.46), and their average tenure in health service was 7.86 years (*SD* = 7.29). Among these participants, 296 were permanent workers (88.36%) and 39 were temporary workers (11.64%). Two hundred and sixty worked full-time (77.61%) and 75 part-time (22.39%). Among these healthcare professionals, 292 worked in public organizations (87.16%), 42 in non-profit organizations (12.54%), and 1 worked in a private organization (0.30%). Finally, 279 worked day shifts (83.30%), 20 worked night shifts (6.00%), and 36 worked both day and night shifts (10.70%).

#### 2.2.2 Measure

All participants completed the 26-item version of the PARTAQUE—see details of the 4 dimensions and the corresponding items above—with the following instructions: “*In your health service…”* They answered with a five-point Likert scale (i.e., 1 = strongly disagree; 5 = strongly agree).

#### 2.2.3 Procedure

An online questionnaire was distributed to healthcare professionals working French establishments. This study was approved by Tours ethical committee and conducted in line with the Helsinki Declaration (World Medical Association, 2013). More precisely, participants received a cover letter explaining to them the study's aims and guaranteeing them of the anonymous, informed, voluntary and confidential nature of the participation.

#### 2.2.4 Statistical analysis and results

Preliminary analyses did not identify any univariate outliers (i.e., |z| > 3.29, *p* < 0.001), in accordance with the recommendations of Tabachnick et al. (2013). Descriptive statistics (Table 1) demonstrated that the kurtosis and skewness coefficients for all items were between −3 and 3 (Kline, 2016). The mean score for the 26 items was 3.30 (range: 2.44–3.96) out of 5.

**Table 1:** Means, standard deviations, and factor loadings of the exploratory factor analysis (Study 1).

	***M***	***SD***	**Skewness**	**Kurtosis**	**IHT**	**TS**	**MPM**	**PA**
ITEM1	3.60	1.16	−0.61	−0.50	0.84			
ITEM2	3.08	1.21	−0.04	−0.95	0.77			
ITEM3	3.60	1.12	−0.50	−0.49	0.70			
ITEM4	3.82	1.03	−0.85	0.49	0.80			
ITEM5	3.96	1.00	−1.00	0.91	0.69			
ITEM6	2.68	1.32	0.29	−1.11		0.58		
ITEM7	3.21	1.19	−0.35	−0.69		0.75		
ITEM8	3.53	1.24	−0.65	−0.52		0.99		
ITEM9	3.23	1.22	−0.31	−0.71		0.82		
ITEM10	3.68	1.23	−0.80	−0.21		0.93		
ITEM11	3.21	1.53	−0.21	−1.45			0.81	
ITEM12	3.47	1.46	−0.55	−1.08			0.83	
ITEM13	3.44	1.36	−0.46	−0.93			0.84	
ITEM14	3.65	1.32	−0.86	−0.33			0.92	
ITEM15	3.60	1.29	−0.79	−0.38			0.92	
ITEM16	3.05	1.24	−0.23	−0.89			0.81	
ITEM17	3.37	1.20	−0.60	−0.44			0.80	
ITEM18	2.88	1.24	0.01	−1.01				0.74
ITEM19	3.24	1.19	−0.31	−0.72				0.77
ITEM20	3.21	1.17	−0.38	−0.56				0.81
ITEM21	3.23	1.15	−0.35	−0.50				0.97
ITEM22	3.46	1.12	−0.61	−0.13				0.89
ITEM23	3.21	1.10	−0.36	−0.28				0.88
ITEM24	2.95	1.13	−0.13	−0.52				0.80
**Eigenvalues**					2.19	1.55	3.30	11.01
**% of variance**					9.13	6.46	13.75	45.88
**α**					0.88	0.92	0.95	0.95

Note. IHT = In-House Training; TS = Team Support; MPM = Multi-Professional Meetings; PA = Project Approach.

We used SPSS version 29 to carry out the Exploratory Factor Analysis (EFA). As positive correlations have been identified between the theoretical sub-dimensions of the PARTAQUE, the principal axis factoring method with an oblique rotation was used (Clark and Watson, 2019). The number of factors to be retained was determined using established methodological guidelines and commonly used criteria, such as the Kaiser-Guttman criterion (Kaiser, 1960), the scree plot (Cattell, 1966), and the proportion of variance explained (Boateng et al., 2018).

After conducting a first EFA, we identified a four-factor solution, which explained 73.16% of the total variance of the 26 items and whose eigenvalues were all >1 (i.e., between 11.75 and 1.63). The four factors corresponded well to the four theoretical sub-dimensions of our construct (i.e., in-house training, team support, multi-professional meetings, and project approach). However, we had to remove two items because they had cross-loadings (Boateng et al., 2018; Clark and Watson, 2019). One for in-house training dimension (i.e., “*Each healthcare professional can suggest topics to be addressed during in-house training*”) and one for team support dimension (i.e., “*Personally, you regularly benefit from team support meetings*”).

Then, we carried out a second EFA, building on identical parameters regarding the rotation. A four-factor solution explaining 75.21% of the total variance of the 24-item version of the PARTAQUE (Table 1) was identified. All eigenvalues were all >1 (i.e., between 11.01 and 1.55), all saturations were above 0.58, and none showed cross-saturation above 0.32 (Boateng et al., 2018). Finally, internal consistency was satisfactory for each factor, with values ranging from 0.88 to 0.95 (Nunnally, 1978). From a theoretical perspective, this second solution was easily interpretable. Indeed, the four-factor model was completely aligned with the four theoretical sub-dimensions of our construct (i.e., in-house training, team support, multi-professional meetings, and project approach). Table 1 presents descriptive statistics of the 24 items, and Table 2 displays inter-item and inter-sub-dimension correlations.

**Table 2:** Inter-item and inter-factor correlations (Study 1).

	**1**	**2**	**3**	**4**	**5**	**TS**	**6**	**7**	**8**	**9**	**10**	**MPM**	**11**	**12**	**13**	**14**	**15**	**16**	**17**	**PA**	**18**	**19**	**20**	**21**	**22**	**23**	**24**
**IHT**						0.45						0.38								0.47							
ITEM1	1	0.75	0.60	0.60	0.53																						
ITEM2		1	0.55	0.56	0.50																						
ITEM3			1	0.51	0.49																						
ITEM4				1	0.84																						
ITEM5					1																						
**TS**						1						0.40								0.65							
ITEM6							1	0.66	0.66	0.54	0.60																
ITEM7								1	0.78	0.67	0.72																
ITEM8									1	0.80	0.88																
ITEM9										1	0.75																
ITEM10											1																
**MPM**												1								0.45							
ITEM11													1	0.74	0.69	0.72	0.70	0.63	0.68								
ITEM12														1	0.77	0.78	0.76	0.64	0.67								
ITEM13															1	0.75	0.73	0.67	0.70								
ITEM14																1	0.92	0.73	0.71								
ITEM15																	1	0.76	0.75								
ITEM16																		1	0.75								
ITEM17																			1								
**PA**																				1							
ITEM18																					1	0.67	0.62	0.69	0.66	0.68	0.71
ITEM19																						1	0.68	0.72	0.71	0.66	0.64
ITEM20																							1	0.81	0.76	0.73	0.67
ITEM21																								1	0.83	0.80	0.74
ITEM22																									1	0.84	0.70
ITEM23																										1	0.78
ITEM24																											1

All correlations are significant at *p* < 0.01. IHT, In-House Training; TS, Team Support; MPM, Multi-Professional Meetings; PA, Project Approach.

### 2.3 Study 2: confirmatory factor analysis of the PARTAQUE

In the second study, the aim was to test the factorial structure of the 24-item version of the PARTAQUE, using a Confirmatory Factor Analysis (CFA). As previously outlined, despite the distinct nature of the 4 key components of the PA, they appear to exhibit commonalities. Indeed, by serving the common objectives of enhancing the quality of communication and, by extension, the quality of the collaboration in the team, they are expected to be mutually reinforcing. Consequently, we hypothesize that the PA—as the global team approach—could be modeled by a second-order factor and its 4 components modeled as first-order factors.

#### 2.3.1 Participants

The new sample comprised 260 French healthcare professionals (36 men and 224 women), including 82 nurses (31.54%), 58 physicians (22.31%), 52 care assistants (20.00%), 24 health managers (9.23%), 4 hospital domestic services aide (1.54%), and 39 other healthcare professionals (15.00%). Their average age was 40.41 years (*SD* = 10.56), and their average tenure in health service was 7.54 years (*SD* = 7.34). Among the participants, 231 were permanent workers (88.85%) and 29 temporary workers (11.15%). One hundred and ninety seven worked full-time (75.77%) and 63 part-time (24.23%). Among these healthcare professionals, 222 worked in public organizations (85.38%), 38 in nonprofit organizations (14.62%), and 0 worked in a private organization (0.00%). Finally, 216 worked day shifts (83.08%), 13 worked night shifts (5.00%), and 31 worked both day and night shifts (11.92%).

#### 2.3.2 Measure

All participants completed the 24-item version of the PARTAQUE with the following instructions: “*In your health service…”* They answered with a five-point Likert scale (i.e., 1 = strongly disagree; 5 = strongly agree).

#### 2.3.3 Procedure

Healthcare professionals working French establishments were invited to complete an online questionnaire. In line with the Helsinki Declaration (World Medical Association, 2013), participants received a cover letter explaining to them the study's aims and guaranteeing them of the anonymous, informed, voluntary and confidential nature of the participation.

#### 2.3.4 Statistical analysis and results

Preliminary analyses did not identify any univariate outliers (i.e., |z| > 3.29, *p* < 0.001), but identified five multivariate outliers [i.e., Mahalanobis distance greater than $\chi_{\left(4\right)}^{2}$ = 18.47, *p* < 0.001] which were excluded, leaving 255 participants for analyses, in accordance with the recommendations of Tabachnick et al. (2013). Descriptive statistics (Table 3) demonstrated that the kurtosis and skewness coefficients for all items were between −3 and 3 (Kline, 2016). The mean score for the 24 items was 3.34 (range: 2.60–3.98) out of 5.

**Table 3:** Means, standard deviations, inter-item and inter-factor correlations (Study 2).

	***M***	***SD***	**Skewness**	**Kurtosis**	**IHT**	**1**	**2**	**3**	**4**	**5**	**TS**	**6**	**7**	**8**	**9**	**10**	**MPM**	**11**	**12**	**13**	**14**	**15**	**16**	**17**	**PA**	**18**	**19**	**20**	**21**	**22**	**23**	**24**
**IHT**	3.62	0.85	−0.67	0.20	0.86						0.42						0.25								0.45							
ITEM1	3.66	1.13	−0.77	−0.18		1	0.71	0.55	0.52	0.50																						
ITEM2	2.99	1.17	−0.06	−0.98			1	0.53	0.51	0.47																						
ITEM3	3.54	1.12	−0.66	−0.35				1	0.54	0.48																						
ITEM4	3.95	0.92	−1.00	1.18					1	0.83																						
ITEM5	3.98	0.90	−0.88	0.80						1																						
**TS**	3.22	1.00	−0.61	0.06							0.90						0.52								0.67							
ITEM6	2.60	1.25	0.19	−1.10								1	0.57	0.56	0.46	0.55																
ITEM7	3.17	1.14	−0.31	−0.57									1	0.75	0.59	0.69																
ITEM8	3.50	1.17	−0.75	−0.08										1	0.76	0.83																
ITEM9	3.23	1.21	−0.46	−0.58											1	0.73																
ITEM10	3.61	1.14	−0.79	0.11												1																
**MPM**	3.46	1.00	−0.77	0.20													0.91								0.54							
ITEM11	3.28	1.41	−0.36	−1.19														1	0.60	0.45	0.66	0.64	0.56	0.57								
ITEM12	3.52	1.36	−0.61	−0.86															1	0.68	0.70	0.66	0.52	0.44								
ITEM13	3.47	1.27	−0.50	−0.76																1	0.62	0.59	0.47	0.50								
ITEM14	3.71	1.19	−0.91	0.14																	1	0.89	0.69	0.61								
ITEM15	3.71	1.14	−0.90	0.28																		1	0.71	0.64								
ITEM16	3.15	1.11	−0.24	−0.61																			1	0.69								
ITEM17	3.42	1.06	−0.63	−0.03																				1								
**PA**	3.10	1.00	−0.54	−0.04																					0.95							
ITEM18	2.82	1.25	0.13	−1.02																						1	0.72	0.60	0.65	0.63	0.61	0.62
ITEM19	3.25	1.20	−0.41	−0.62																							1	0.69	0.74	0.74	0.70	0.62
ITEM20	3.16	1.17	−0.37	−0.59																								1	0.79	0.82	0.78	0.67
ITEM21	3.17	1.12	−0.45	−0.46																									1	0.85	0.79	0.74
ITEM22	3.37	1.13	−0.68	−0.08																										1	0.84	0.69
ITEM23	3.11	1.09	−0.34	−0.35																											1	0.79
ITEM24	2.84	1.06	−0.29	−0.63																												1

All correlations are significant at *p* < 0.01. Alpha coefficients for each subdimension are in orthogonal rows. IHT = In-House Training; TS = Team Support; MPM = Multi-Professional Meetings; PA = Project Approach.

A series of CFA with the Maximum Likelihood (ML) estimation method was then carried out using AMOS version 25. To assess the fit of the models, we used the following indices: Standardized Root Mean Square Residual—SRMR (≤ 0.05 good fit), Root Mean Square Error of Approximation—RMSEA (≤ 0.05 excellent fit, ≤ 0.08 adequate fit), Comparative Fit Index—CFI (≥0.95 excellent fit, ≥0.90 adequate fit), and Akaike Information Criterion—AIC, which should be as low as possible (Kline, 2016).

**Figure 1:**
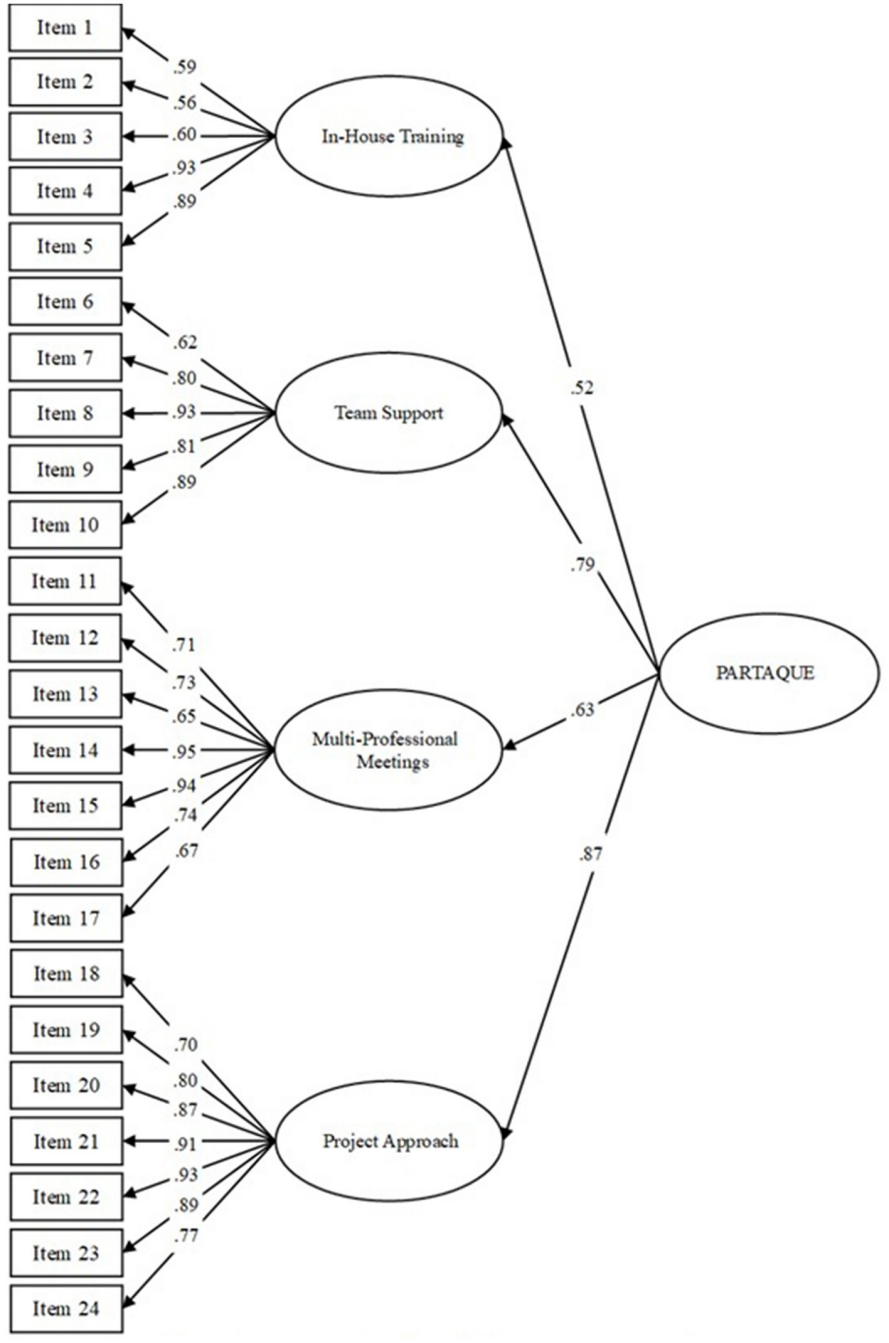
CFA with second-order factor model (Study 2). All standardized coefficients are significant at *p* < 0.001.

We carried out two factorial models. The first model tested included four first-order factors (i.e., in-house training, team support, multi-professional meetings, and project approach). Results indicate a good fit of this theoretical model to the data [χ^2^ = 645.17 (241), *p* < 0.001; SRMR = 0.06; RMSEA = 0.08; CFI = 0.92; AIC = 811.17]. The second model carried out included a second-order factor (i.e., PARTAQUE). Results reveal that data also fitted this model well [χ^2^ = 646.68 (243), *p* < 0.001; SRMR = 0.06; RMSEA = 0.08; CFI = 0.92; AIC = 808.68]. Finally, the comparison between these two models indicates no significant difference between the two models [$\chi_{\left(2\right)}^{2}$ = 1.51, *p* > 0.05]. However, the second-order factor model (Figure 1) was retained because it was more consistent with the theoretical nature of our construct, reflecting the PA as the second-order factor and all sub-dimension—also called components and in the initial PA description of the article, as first-order factors.

### 2.4 Study 3: convergent and predictive validity of PARTAQUE

The aim of the third study was to examine the convergent validity and predictive validity of the PARTQUE. We did so by performing bivariate Pearson correlations.

#### 2.4.1 Participants

The sample consisted of 595 French healthcare professionals (95 men and 500 women), including 196 nurses (32.94%), 132 physicians (22.18%), 118 care assistants (19.83%), 67 health managers (11.26%), 9 hospital domestic services aide (1.51%), and 72 other healthcare professionals (12.10%). Their average age was 41.06 years (*SD* = 10.51), and their average tenure in health service was 7.72 years (*SD* = 7.31). Among the participants, 527 were permanent workers (88.57%) and 68 temporary workers (11.43%). Four hundred and fifty seven worked full-time (76.81%) and 138 part-time (23.19%). Among these healthcare professionals, 514 worked in public organizations (86.39%), 80 in nonprofit organizations (13.45%), and 1 worked in a private organization (0.17%). Finally, 495 worked day shifts (83.19%), 33 worked night shifts (5.55%), and 67 worked both day and night shifts (11.26%).

#### 2.4.2 Measure

Participants completed the 10 scales (i.e., participative approach, participative decision-making, communication, general health, voice behaviors, work engagement, thriving at work, job satisfaction, emotional exhaustion, person-service fit) using a five-point Likert scale (i.e., 1 = strongly disagree; 5 = strongly agree), except for general health (i.e., 1 = very poor; 5 = excellent).

**The Participative Approach** was measured with the 24-item version of the PARTAQUE with the following instructions: “*In your health service…”* Analyses revealed good internal consistency for in-house training (α = 0.87), team support (α = 0.91), multi-professional meetings (α = 0.94), project approach (α = 0.95), and the total scale (α = 0.95).

**Participative Decision-Making** were assessed with the sub-dimension Practices of Participative Decision-Making of Virtuous Organizational Practices inventory (Aubouin-Bonnaventure et al., 2021) composed of 3 items (e.g., “In my health service, decisions are made collectively”). The internal consistency was satisfactory (α = 0.90).

**Communication** was measured with the sub-dimension Communication Practices of Virtuous Organizational Practices inventory (Aubouin-Bonnaventure et al., 2021) composed of 3 items (e.g., “My health service provides time for information sharing.”). The internal consistency was satisfactory (α = 0.85).

**Voice Behaviors** were measured with Botero and Van Dyne's (2009) scale, including 6 items (e.g., “I get involved in issues that affect the quality of life in my health service”). The internal consistency was satisfactory (α = 0.89).

**Work Engagement** was assessed with Schaufeli et al.'s (2017) scale composed of 3 items (e.g., “At my work, I feel bursting with energy”). The internal consistency of this scale was satisfactory (α = 0.73).

**Thriving at Work** was measured with Porath et al.'s (2012) scale, including 10 items: 5 for learning (e.g., “I continue to learn more as time goes by”), and 5 for vitality (e.g., “I feel alive and vital”) and. Two items were reversed (i.e., “I am not learning” and “I do not feel very energetic”). The internal consistency was satisfactory (α = 0.71).

**Job Satisfaction** was assessed using the item of Tavani et al. (2014); i.e., “Overall, I am satisfied with my work”).

**Emotional Exhaustion** was measured with Lapointe et al.'s (2011) scale, composed of 5 items (e.g., “‘I feel emotionally drained by my work”). The internal consistency was satisfactory (α = 0.89).

**Person-Service Fit** was assessed with Saks and Ashforth's (1997) scale including 3 items (e.g., “Your health service measure up to the kind of health service you were seeking”). Analyses revealed good internal consistency (α = 0.90).

**Intention to Stay** was measured using the item of Cho et al. (2009); “I am likely to leave this service within the next 12 months.”

**General Health** was assessed with the single item of Krause and Jay (1994) (i.e., “Overall, how would you rate your health?”). Participants completed a five-point Likert scale (i.e., 1 = very poor; 5 = excellent).

#### 2.4.3 Procedure

An online questionnaire for healthcare professionals was distributed to healthcare facilities in France. All participants received a cover letter explaining to them the study's aims and guaranteeing them of the anonymous, informed, voluntary and confidential nature of the participation. Thus, this study was conducted in line with the Helsinki Declaration (World Medical Association, 2013).

#### 2.4.4 Statistical analysis and results

Analyses did not identify any (i.e., |z| > 3.29, *p* < 0.001) and 5 multivariate outliers [i.e., Mahalanobis distance greater than $\chi_{\left(14\right)}^{2}$ = 36.12, *p* < 0.001] which were excluded, leaving 590 participants for the analyses (Tabachnick et al., 2013). Descriptive statistics (Table 4) revealed that the kurtosis and skewness coefficients for all items were between −3 and 3 (Kline, 2016).

**Table 4:** Descriptive statistics and correlations (Study 3).

		**M**	**SD**	**Skewness**	**Kurtosis**	**1**	**2**	**3**	**4**	**5**	**6**	**7**	**8**	**9**	**10**	**11**	**12**	**13**	**14**	**15**
**1**	PARTAQUE (global)	3.36	0.78	−0.51	0.26	1	0.69	0.75	0.83	0.84	0.56	0.64	0.24	0.48	0.34	0.46	0.40	−0.36	0.47	0.24
**2**	In–House Training	3.61	0.88	−0.56	0.07		1	0.33	0.44	0.46	0.33	0.45	0.21	0.37	0.20	0.37	0.30	−0.26	0.37	0.17
**3**	Multi–Professional Meetings	3.42	1.11	−0.74	−0.14			1	0.45	0.48	0.45	0.48	0.16	0.39	0.29	0.32	0.28	−0.25	0.34	0.16
**4**	Team Support	3.25	1.05	−0.54	−0.23				1	0.66	0.44	0.48	0.19	0.33	0.26	0.37	0.32	−0.30	0.34	0.18
**5**	Project Approach	3.14	1.00	−0.45	−0.10					1	0.50	0.57	0.19	0.39	0.29	0.38	0.35	−0.30	0.39	0.22
**6**	Participative decision–making	2.92	1.07	−0.11	−0.65						1	0.81	0.27	0.38	0.30	0.38	0.38	−0.33	0.42	0.19
**7**	Communication	3.33	1.00	−0.43	−0.26							1	0.30	0.46	0.30	0.44	0.43	−0.37	0.48	0.25
**8**	General health	3.34	0.79	0.13	0.21								1	0.16	0.26	0.41	0.39	−0.48	0.32	0.20
**9**	Voice behaviors	3.81	0.71	−0.65	1.64									1	0.34	0.39	0.22	−0.20	0.31	0.16
**10**	Work engagement	5.03	0.99	−0.63	1.13										1	0.54	0.51	−0.38	0.53	0.37
**11**	Thriving at work	3.37	0.51	−0.56	0.80											1	0.64	−0.57	0.61	0.37
**12**	Job satisfaction	3.79	0.88	−0.95	1.33												1	−0.60	0.62	0.39
**13**	Emotional exhaustion	2.86	0.98	0.22	−0.59													1	−0.52	−0.41
**14**	Person–service fit	3.80	0.88	−0.79	0.70														1	0.55
**15**	Intention to stay	4.07	1.14	−1.23	0.81															1

All correlations are significant at *p* < 0.001.

#### 2.4.5 Correlations

First, analyses of convergent validity demonstrated significantly positive correlations between PARTAQUE on the one hand, and participative decision-making (*r* = 0.56, *p* < 0.001) and communication (*r* = 0.64, *p* < 0.001) on the other (Table 4). Second, analyses for predictive validity revealed that PARTAQUE was significantly and positively associated with general health (*r* = 0.24, *p* < 0.001), voice behaviors (*r* = 0.48, *p* < 0.001), work engagement (*r* = 0.34, *p* < 0.001), thriving at work (*r* = 0.46, *p* < 0.001), job satisfaction (*r* = 0.40, *p* < 0.001), person-service fit (*r* = 0.47, *p* < 0.001), and intention to stay (*r* = 0.24, *p* < 0.001).

## 3 Discussion

The PA is a work-organization model designed to improve the quality of patient care through the enhancement of the quality of work life for caregivers. Of note that the implementation of this teamwork model has been legally mandatory in France since 2004, guaranteeing its integration into all palliative care teams nationwide.

The aim of this study was to develop and validate a new scale to ensure a scientifically validated measure of the PA, a widespread service organization in the French palliative departments. On the basis of three independent studies, we developed a 24-item scale with satisfactory psychometric properties that reflects well the theoretical nature of our concept. As such, the second-order factorial structure appropriately reflects the distinction between the PA—a global department organizational model, posited as a second-order construct, and its sub-dimensions (i.e., in-house training, team support meetings, multi-professional meetings, project approach)—posited as first-order constructs. In addition, the convergent and predictive validity analyses demonstrated significant relationships in the expected direction with variables from the nomological network of the PA (e.g., communication, general health, voice behaviors).

Although the PA has been around for more than 20 years in the field of oncology and palliative care, and that several studies have measured its impact on various work outcomes, there has been no scientifically validated tool to measure it as a distinct construct. Therefore, this research provides an important contribution to current knowledge by providing a ready-to-use, rigorous tool that enables both researchers and practitioners to better measure and monitor PA in cancer teams.

From a scientific standpoint, the development and initial validation of the PARTAQUE offer interesting avenues for research targeting for work-organization models in healthcare. Indeed, it allows for a concomitant robust measure of key organizational determinants (e.g., communication structures and practices, team training, project management approach) and highlights its positive relations with important work outcomes, such as health at work indicators and performance proxies. Moreover, given that this scale was developed within a particular occupational context (i.e., healthcare teams), it facilitates a more comprehensive understanding of the intertwined factors that influence the quality of work life for caregivers and consequently the quality of care.

From a practical perspective, the use PARTAQUE allows for a rigorous preliminary diagnosis prior to the implementation of the PA in a given team. This could potentially allow managers and heads of departments to develop a more nuanced understanding of which aspects of the PA might be worth prioritizing in terms of structural and human resources allocations. It is also worth considering that this could be an important tool for making informed decisions regarding the sequence of the implementation of the components of the PA. Furthermore, upon the full implementation of the PA, the introduction of a longitudinal measure of the PARTAQUE and its associated health and work outcomes could prove advantageous for the purpose of monitoring the quality of work life experienced by caregivers and the quality of care they provide. This would establish the foundation for targeted managerial interventions concerning the various components of the PA. Moreover, at a more macro level, the existence of a validated scale of the PA would provide the Ministry of Health, the political leaders, and the directors of healthcare institutions with a precise national map of the realities on the front line regarding the implementation of a service organization which they have promoted and even enshrined in a legal framework.

Despite the implementation of a meticulous and stringent procedure for scale development and validation, our research is inherently constrained by its intrinsic limitations, akin to the limitations of any research endeavor. First, the cross-sectional research design of the third study impediments our ability to infer causal relationships between the PA and the measured constructs. This raises the concern of common method bias (Podsakoff et al., 2003) that could be addressed by conducting longitudinal or quasi-experimental studies to identify potential causal relationships between these the PA and the theorized outcomes. Secondly, given that all three studies are reliant upon French samples, the cultural invariance of the PARTAQUE may be subject to scrutiny (Zhou et al., 2019). Consequently, subsequent research endeavors may benefit from extending the validation procedure (e.g., test of divergent validity, test of temporal invariance) and conduct more in-depth factorial analysis (e.g., ESEM and Bi-ESEM models). In addition, it could be interesting to assess its relevance and robustness in varied cultural contexts, which exhibit notable similarities in the configuration of healthcare departments (e.g., the province of Québec, Canada).

## 4 Conclusion

This study provides a validated and ready-to-use scale to measure and monitor the PA in cancer teams. We believe that the PARTAQUE will be beneficial for healthcare managers and heads of departments in their efforts to create positive work environments that supports both the quality of work life of caregivers and the quality of care they provide.

## Data Availability

The original contributions presented in the study are included in the article/supplementary material, further inquiries can be directed to the corresponding author.

## Footnotes

^1^Circular n° 257 of the 9^th^ of June 9, 2004; France's Ministry of Health and Social Protection.
