# Correction: Development and validation of the participatory approach questionnaire (*PARTAQUE*) in cancer teams: a ready-to-use tool for enhancing the quality of work life for caregivers

**DOI:** 10.3389/fpsyg.2025.1682372

**Published:** 2025-08-29

**Authors:** Frontiers Production Office

**Affiliations:** Frontiers Media SA, Lausanne, Switzerland

**Keywords:** Participative Approach (PA), cancer teams, scale validation, quality of work life, quality of care

Due to an error during the finalisation of the article, shared first authorship was not denoted for authors Julien Lejeune and Anaïs Galy.

The corrected author details featuring the shared first authorship notation are shown below.

Julien Lejeune^1,2^^*†^, Anaïs Galy^1,3†^, Evelyne Fouquereau^1^, Christine Jeoffrion^1,4^, Julia Aubouin-Bonnaventure^1^, Hélène Coillot^5^, Jimmy Bordarie^1^, Romuald Seizeur^6^ and Philippe Colombat^1^

^1^QualiPsy UR 1901, Psychology Department, University of Tours, Tours, France

^2^Service d'onco-hématologie pédiatrique, CHRU of Tours, Tours, France

^3^Pôle Santé HEC Montréal, Department of Human Resources, HEC Montréal, Montréal, QC, Canada

^4^LIP/PC2S, Université de Grenoble Alpes, Université Savoie Mont Blanc, Grenoble, France

^5^EE 1901 Quality of Life and Psychological Health, Université de Tours, Tours, France

^6^Service de Neurochirurgie, CHU Brest, Brest, France

^†^These authors share first authorship

The original version of this article has been updated.

